# Real-World Study on Vedolizumab Serum Concentration, Efficacy, and Safety after the Transition from Intravenous to Subcutaneous Vedolizumab in Inflammatory Bowel Disease Patients: Single-Center Experience

**DOI:** 10.3390/ph16020239

**Published:** 2023-02-05

**Authors:** Vlasta Oršić Frič, Vladimir Borzan, Ines Šahinović, Andrej Borzan, Sven Kurbel

**Affiliations:** 1 Faculty of Medicine, J. J. Strossmayer University of Osijek, 31000 Osijek, Croatia; 2 Department of Clinical Laboratory Diagnostics, University Center Hospital Osijek, 31000 Osijek, Croatia; 3 Poliklinika Aviva, 10000 Zagreb, Croatia

**Keywords:** inflammatory bowel disease, vedolizumab, treatment outcome

## Abstract

Little is known about how the change from intravenous to subcutaneous vedolizumab in a real-life setting in inflammatory bowel disease patients on stable maintenance therapy affects clinical outcomes. We compared the data on vedolizumab serum trough concentration, efficacy, and safety prior to and six months after the switch from intravenous to subcutaneous vedolizumab. In total, 24 patients, 13 with ulcerative colitis (UC) and 11 with Crohn’s disease (CD), were included. Mean serum trough concentration of intravenous vedolizumab was significantly lower than mean serum trough concentration of subcutaneous vedolizumab (*p* = 0.002). There was no significant difference between C-reactive protein levels, fecal calprotectin levels or clinical scores (Harvey–Bradshaw index or Partial Mayo score) prior to transition to subcutaneous vedolizumab and after 6 months. In four (16.7%) patients, two CD and two UC, therapy was discontinued during the follow-up period with a median of 5 months (minimum–maximum: 4–6). In all patients, therapy was discontinued due to loss of response. In total, 13 adverse events were reported by 11 patients, and the most common adverse event was COVID-19. No serious adverse events were reported. In conclusion, subcutaneous vedolizumab has shown to be effective and safe in patients on previously established maintenance therapy with intravenous vedolizumab.

## 1. Introduction

Crohn’s disease (CD) and ulcerative colitis (UC) are chronic inflammatory diseases of the digestive tract, and anti-inflammatory drugs, whether conventional or biologics, have a central role in their treatment [1,2]. Vedolizumab is approved for the induction and maintenance of remission in patients with CD and UC. It is a humanized monoclonal antibody which binds to α4β7 protein on the surface of helper T lymphocytes and prevents its binding to mucosal vascular addressin cell adhesion molecule 1 (MadCAM1) on endothelial cells of blood vessels in the intestinal wall. This prevents lymphocyte migration and inflammatory response in the intestinal wall [3,4]. In Croatia, vedolizumab was approved in 2016 as a drug for intravenous application. From June 2021, a subcutaneous form of vedolizumab was approved as a maintenance therapy after the induction with at least two intravenous doses. In VISIBLE 1 and VISIBLE 2 studies, subcutaneous vedolizumab has proven to be more effective than placebo in the treatment of moderately to severely active UC and CD [5,6]. In both studies, subcutaneous vedolizumab was studied as a maintenance therapy in patients who responded to two intravenous doses of vedolizumab 300 mg at week 0 and 2. The primary endpoint was clinical remission in week 52. In VISIBLE 1, 46.2% of UC patients on subcutaneous vedolizumab achieved clinical remission vs. 14.3% of patients on placebo (*p* < 0.001). In VISIBLE 2, clinical remission at week 52 was achieved in 48.0% of CD patients receiving subcutaneous vedolizumab compared to 34.3% of patients on placebo (*p* = 0.008). Moreover, in the VISIBLE 1 study, the efficacy of the subcutaneous and intravenous forms of vedolizumab has proven to be comparable, as clinical remission at week 52 was achieved in 42.6% of patients receiving intravenous vedolizumab. In both studies, subcutaneous vedolizumab has proven to be safe and tolerable, with a safety profile comparable to that of intravenous vedolizumab (except for injection site reactions) [5,6].

Therapeutic drug monitoring (TDM) is used regularly in the treatment of patients with anti-tumor necrosis factor alpha (anti-TNF) drugs, but its role in treatment with newer biologics, including vedolizumab, is not completely clear [7]. Although pharmacokinetic data show that vedolizumab serum concentrations during induction and maintenance therapy correlate with clinical outcomes, it is probably not as evident as for anti-TNF drugs [7]. Moreover, there is no clear cut-off for vedolizumab serum concentration, neither for induction nor for maintenance therapy, with which positive clinical outcomes would be associated [8]. In the VISIBLE 1 clinical study, average steady state vedolizumab trough concentration in patients with UC treated with subcutaneous vedolizumab was 35.8 mg/L (SD ± 15.2), which is comparable to average vedolizumab through concentration for intravenous dosing every 4 weeks [9]. It has been shown, for both subcutaneous and intravenous vedolizumab, that with higher serum trough concentrations, more patients achieve clinical remission and endoscopic response [10].

Little is known about how the change from intravenous to subcutaneous vedolizumab in patients that are on stable maintenance therapy affects clinical outcomes. Recent real-world studies [11,12,13,14] showed that clinical outcomes did not significantly change in patients who changed to subcutaneous vedolizumab, but more data are still needed. In this study, we present data on vedolizumab serum concentration, efficacy, and safety in patients that transitioned from maintenance therapy with intravenous vedolizumab to the subcutaneous form of the drug.

## 2. Results

**Figure 1:**
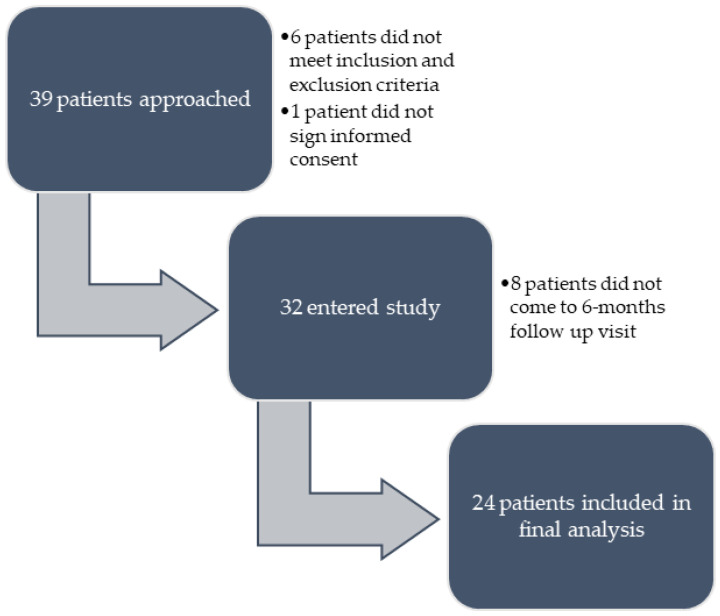
Algorithm of patients’ enrolment in the study.

Thirty-two patients entered the study, but only patients that finished the 6-month visit were included in the further analysis. The study’s algorithm is shown in Figure 1. In total, 24 patients, 13 with UC and 11 with CD, were included in the final analysis. Among CD patients, most patients had an inflammatory phenotype and ileocolonic localization, and among UC patients, most had left-sided colitis. A little over half of the patients were biologic-naïve prior to the beginning of the vedolizumab therapy (54.2%). Only one patient was taking corticosteroids (prednisone) at the time of transition to subcutaneous vedolizumab, but it was at a low dose and due to arthritis and not inflammatory bowel disease (IBD). Patients’ baseline characteristics are presented in Table 1.

**Figure 2:**
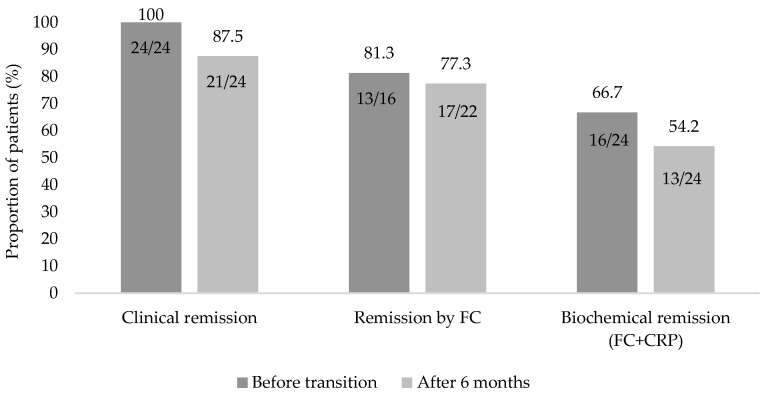
Proportion of patients in clinical remission, remission defined by fecal calprotectin (FC) < 250 µg/g, and biochemical remission, defined as C-reactive protein (CRP) ≤ 5 mg/L and/or FC < 250 µg/g, prior to transition to subcutaneous vedolizumab and after the transition.

Mean steady-state serum trough concentration of intravenous vedolizumab was significantly lower than mean steady-state serum trough concentration of subcutaneous vedolizumab (*p* = 0.002) (Table 2). There was no significant difference between median C-reactive protein (CRP) and fecal calprotectin values or clinical scores (Harvey–Bradshaw index, HBI, or Partial Mayo score, PMS) prior to transition to subcutaneous vedolizumab and 6 months after the transition (Table 2). All patients were in clinical remission at the time of transition to subcutaneous vedolizumab, and 16 (66.7%) were in biochemical remission prior to transition. At follow-up, 87.5% (21/24) of patients were in clinical, and 54.2% (13/24) were in biochemical remission. In five patients who had fecal calprotectin >250 µg/g at follow-up, endoscopy was performed to confirm relapse. Endoscopic signs of disease flare were found in three patients (12.5%). There was no significant difference in the proportion of patients in clinical remission, biochemical remission or remission defined by fecal calprotectin <250 µg/g before and after the transition to subcutaneous vedolizumab (*p* = 0.250, *p* = 0.453, and *p* = 1.000, respectively; Figure 2). In four (16.7%) patients, two CD and two UC, therapy was changed during the follow-up period with a median of 5 months (minimum–maximum 4–6) from the transition to subcutaneous vedolizumab. In all patients, therapy was changed due to the loss of response. In two CD patients, vedolizumab dosing was optimized to every 4 weeks intravenously, and two UC patients started corticosteroids and were planned to switch to another biologic. No patient had surgery or was hospitalized due to IBD during the follow up period.

In total, 13 (54.2%) treatment-emergent adverse events (TEAE) were reported by 11 patients, and the most common TEAE was COVID-19 (Table 3). One patient reported injection site reaction (erythema around the injection site), and no serious adverse events were reported.

## 3. Discussion

Subcutaneous vedolizumab is a novel formulation of vedolizumab that can be used as a maintenance therapy in IBD patients as an alternative to the intravenous formulation. It is administered in a dosage of 108 mg every other week, compared to a dosing scheme of 300 mg every 8 weeks (or 4 weeks in optimized dosing) for intravenous formulation. Subcutaneous therapy has many advantages over intravenous administration of drugs. It requires less frequent visits to the hospital, and it is less time-consuming and therefore more convenient for patients. It also reduces the use of staff resources and the financial burden on a healthcare system. Subcutaneous therapy can also have some disadvantages, such as less control over patient’s adherence to the therapy or potential local allergic reactions. As well, there is a possibility of an inappropriate storage of the drug at home, which can lead to lower effectiveness or adverse events. Some patients, moreover, dislike the idea of self-injecting or more frequent dosing of subcutaneous formulations [15]. In recent studies on patient acceptance of switching from intravenous infliximab or vedolizumab to subcutaneous formulations of the drug, the majority (58–78%) of the patients accepted the switch, and the main motivation was saving time [16,17].

Phase III trials, VISIBLE 1 and 2, presented data on the efficacy and safety of subcutaneous vedolizumab in patients with UC and CD in a controlled setting of a clinical trial. Our real-world study is assessing vedolizumab serum trough concentration, efficacy, and safety after the transition from intravenous to subcutaneous vedolizumab in patients that were on a prior stable maintenance therapy with intravenous vedolizumab. Median duration of prior intravenous vedolizumab therapy in our cohort is 11 months, with some patients receiving intravenous vedolizumab for almost 5 years (maximum of 58 months) prior to transition.

In our cohort, mean steady state serum vedolizumab trough concentration increased from 22.86 mg/L (±15.66 mg/L) for intravenous vedolizumab to 35.62 mg/L (±15.46 mg/L) for subcutaneous vedolizumab. Higher serum vedolizumab trough concentration after the transition to subcutaneous vedolizumab is comparable to data from the VISIBLE 1 and VISIBLE 2 studies. Initial pharmacokinetic modelling in the VISIBLE 1 trial estimated median serum trough concentration for subcutaneous vedolizumab at 34.6 mg/L (90% CI, 15.5–72.8 mg/L) [5]. In the VISIBLE 2 trial, median serum trough concentration at week 52 was 30.2 mg/L (0.78–70.1 mg/L) [6]. Our results are also in line with published real-world data. Volkers et al. [11] reported median serum vedolizumab trough concentration of 36 mg/L (IQR 29–39 mg/L) 24 weeks after transition from intravenous vedolizumab, although data on trough concentration were available from only eight patients. In the study by Wiken et al. [12], median vedolizumab plasma concentration was even higher, at 44 mg/L (IQR 28.9–64.7), but some patients were on a shorter dosing interval than the recommended 2-week dosing, and it is not evident whether the reported concentration is trough concentration. Two other studies [13,14] reported lower serum vedolizumab trough concentration in patients on subcutaneous vedolizumab (22.7 mg/L and 19 mg/L, respectively); in both, however, a comparable increase in serum vedolizumab trough concentration was seen after the switch from intravenous vedolizumab (Δ12.7 mg/L and Δ10.9 mg/L, compared to Δ12.8 mg/L in our study). As vedolizumab concentration in our and other real-life studies was measured using different ELISA-assays, differences in absolute values are probably due to low reproducibility and accuracy of the method. Although studies consistently show elevated trough levels of subcutaneous vedolizumab, the clinical significance of higher trough concentrations is not yet clear. Subcutaneous drugs have lower bioavailability, lower peak concentrations, and differences between peak and trough concentrations are smaller [18]. Therefore, two formulations cannot be directly compared. Total drug exposure for intravenous 8-week dosing and subcutaneous 2-week dosing of vedolizumab is shown to be similar [9], so increased trough levels for subcutaneous formulation are not expected to lead to better clinical outcomes. Further studies are needed to confirm or dismiss this statement. The exposure–response relationship was observed in studies for both formulations of vedolizumab [10,19,20]; however, exact cut-off values of vedolizumab trough levels for achieving positive clinical outcomes are still unknown [21].

A study by Ventress et al. [13] reported a significant, but not clinically important, rise in fecal calprotectin 12 weeks after the transition from intravenous vedolizumab. On the contrary, Bergqvist et al. [14] showed a decrease in median fecal calprotectin in all cohort subjects and in CD patients after the drug application type switch. In our patients, there was no significant difference between CRP, fecal calprotectin, or clinical scores prior to and after the transition to subcutaneous vedolizumab. Our results also showed that there was no significant difference in the proportion of patients in clinical and biochemical remission before and after the switch, implicating that patients on established vedolizumab therapy could be switched to subcutaneous vedolizumab without compromising the drug’s efficacy. Similar results were shown by two other real-life studies [11,12] further confirming the previous statement. During the 6 months follow-up, four patients (16.7%) discontinued subcutaneous vedolizumab because of loss of response, showing that drug persistence in our study is slightly lower than in patients on long-term maintenance therapy with intravenous vedolizumab [22,23]. In patients who switched to subcutaneous vedolizumab in other real-life studies, drug persistence was also slightly higher than in our cohort (88.1–95.5% at 6 months) [11,14].

No serious adverse events were reported during the follow-up, with COVID-19 being the most reported adverse event by patients. Injection site reactions are probably underreported due to predominantly mild reactions, as only one patient reported mild erythema around an injection site.

The main limitation of this study is the low number of patients included. However, as there are only a few real-world studies published so far, it will add valuable data to overall knowledge on efficacy and safety of subcutaneous vedolizumab as a maintenance therapy in a real-world setting, especially in patients that are already on established vedolizumab therapy. Due to the low number of patients included, we did not group our results according to diagnosis (CD and UC); instead, data are presented collectively. Moreover, there is no control group, as the study was designed to follow a cohort of patients prior to and after the switch to subcutaneous vedolizumab. Another potential limitation of this study is that we did not present data on vedolizumab immunogenicity, as there is no commercially available kit for detection of antidrug antibodies for vedolizumab. According to data from previous studies, immunogenicity of vedolizumab is low, so we do not expect that this data would be of clinical importance. Finally, endoscopy data prior to and after the switch were not presented, as only a few patients had data on endoscopy findings available.

## 4. Materials and Methods

### 4.1. Patients and Protocol Description

We approached CD and UC patients treated with vedolizumab at the Department of Gastroenterology and Hepatology in the University Hospital Centre Osijek. At our department, all patients that had a stable disease (based on clinical symptoms, fecal calprotectin, or endoscopy) and were not on corticosteroid therapy due to IBD or optimized vedolizumab dosing (every 4 weeks), were planned to transition to a subcutaneous form of vedolizumab after its approval in Croatia. Inclusion criteria for this study were: signed informed consent, diagnosis of IBD, age > 18 years, transition from intravenous to subcutaneous vedolizumab (prior or current), and at least four intravenous doses of vedolizumab received. Exclusion criteria were unwillingness to sign informed consent and continuation of intravenous vedolizumab. Data on age, gender, body mass, diagnosis, previous therapy for IBD, previous surgery due to IBD, comorbidities, concomitant therapy, CRP (at the time of the last intravenous dose of vedolizumab), HBI or PMS were gathered either from patients or from the electronic medical records. In patients that were still on intravenous vedolizumab, on the day of infusion, blood was taken for serum vedolizumab trough level. If patients were included in the study after they had already transitioned to subcutaneous vedolizumab, serum trough level of intravenous vedolizumab was taken from their medical records. The fecal calprotectin level was also collected from patients’ medical records if it was recorded within six months prior to transition to subcutaneous vedolizumab, and only values from the Department of Laboratory Diagnostics of the University Hospital Centre Osijek were considered valid.

Patients were followed prospectively for 6 months from transition to subcutaneous vedolizumab or until a change in therapy. Data on a change in therapy (introduction of corticosteroids, vedolizumab optimization to every 4 weeks intravenously, or vedolizumab cessation), hospitalizations, surgery due to IBD, TEAE, CRP, and fecal calprotectin level, were taken at the 6-month visit. Data for HBI or PMS calculation were also taken. A four-week window was allowed around this timeline, as it was a real-world study. On the day of the dosing, blood was collected for subcutaneous vedolizumab serum trough level.

### 4.2. Outcomes

The primary outcome in this study was the change in serum vedolizumab trough level after the transition from intravenous to subcutaneous vedolizumab. The secondary outcomes were proportion of patients with change of therapy, change in CRP and fecal calprotectin level, change in clinical scores (HBI or PMS), remission rate, rate of hospitalizations or surgery due to IBD, and TEAE incidence after the transition to subcutaneous vedolizumab.

A change in therapy was defined as vedolizumab cessation, change to intravenous vedolizumab with a dosing schedule of every 4 weeks, or initiation of corticosteroids, due to loss of response or an adverse event. Clinical remission was defined as HBI < 5 and PMS < 2, and biochemical remission as CRP level ≤ 5 mg/L and/or fecal calprotectin < 250 µg/g [24,25]. TEAE were presented by system organ class and preferred term (Medical Dictionary for Regulatory Activities, MedDRA, version 25.1).

### 4.3. Vedolizumab Serum Trough Concentration

For vedolizumab serum concentration venous blood was sampled in a 4 mL tube without anticoagulant (BD Vacutainer, Becton, Dickinson, and Company, Franklin Lakes, NJ, USA). All blood samples for vedolizumab serum trough concentration were processed at the Department of Laboratory Diagnostics of University Hospital Centre Osijek. A blood sample from each patient was centrifuged for 10 min at 1370× *g*, and 2 mL of serum was separated and stored at −20 °C until analysis. In the serum sample, vedolizumab trough concentration was measured using a Promonitor VDZ sandwich enzyme-linked immunosorbent assay (ELISA) method (Progenika Biopharma S.A., Grifols, Barcelona, Spain) according to the manufacturer’s protocol on an ELISA processor ETI-Max 3000 (DiaSorin S.p.A, Saluggia, Italy).

### 4.4. Statistics

Categorical variables are presented with absolute and relative frequencies. Numerical variables are presented as mean and standard deviation in case of normal distribution, or as a median and min-max range if data were not normally distributed. Differences between numerical variables of two dependent groups were calculated using paired Student’s *t*-test or Wilcoxon test. Missing data are shown by presenting numbers of data points included in analysis in figures and tables. Differences between categorical variables were calculated using McNemar’s test. All testing was two-tailed, and *p* < 0.05 was considered statistically significant. For statistical analysis, the MedCalc program was used (version 17.9.0, MedCalc Software, Osted, Belgium).

### 4.5. Ethical Considerations

The study was performed in accordance with the principles of the Declaration of Helsinki. The study was approved by the Ethics Committee of the Faculty of Medicine of the J. J. Strossmayer University of Osijek (602-04/22-08/02, 30 April 2022). Patients that agreed to participate in this study were included after they had signed the informed consent.

## 5. Conclusions

In conclusion, subcutaneous vedolizumab has been shown to be effective and safe in patients on previously established maintenance therapy with intravenous vedolizumab. Higher trough concentration of subcutaneous vedolizumab in our cohort did not lead to significant changes in clinical scores, CRP or fecal calprotectin level; however, further research is needed to establish whether it can lead to better clinical outcomes.

## Figures and Tables

**Table 1:** Baseline patients’ characteristics.

Patient Characteristics (N = 24)	
**Female**, n (%)	8 (33.3)
**Age**, years, median (minimum, maximum)	50 (25, 77)
**Body mass**, kilograms, median (minimum, maximum)	85 (55, 116)
**Currently smoking**, n (%)	2 (8.3)
**Diagnosis**, n (%)	
CD	11 (45.8)
UC	13 (54.2)
**Duration of IV vedolizumab therapy**, months, median (minimum, maximum)	11 (5–58)
**Age at onset, CD**, N = 11 (n, %)	
A1: <16 years	1 (9.1)
A2: 17–40 years	5 (45.45)
A3: >40 years	5 (45.45)
**Disease location of CD**, N = 11 (n, %)	
L1: Ileal	2 (18.2)
L2: Ileocolonic	7 (63.6)
L3: Colonic	2 (18.2)
L4: Upper gastrointestinal tract	1 (9.1)
**CD behaviour**, N = 11 (n, %)	
B1: Inflammatory	7 (63.6)
B2: Stricturing	3 (27.3)
B3: Fistulizing	1 (9.1)
P: Perianal disease	1 (9.1)
**Age at onset, UC**, N = 13 (n, %)	
A1: <16 years	0 (0)
A2: 17–40 years	6 (46.2)
A3: >40 years	7 (53.8)
**Disease location of UC**, N = 13 (n, %)	
E1: Proctitis	0 (0)
E2: Left-sided colitis	8 (61.5)
E3: Extensive colitis	5 (38.5)
**Therapy prior to beginning of IV vedolizumab**, n (%)	
Biologic-naïve	13 (54.2)
ASA, corticosteroids	8 (33.3)
AZA, MTX	5 (20.8)
Biologic-experienced	11 (45.8)
1	5 (20.8)
2 or more	6 (25.0)
**Prior surgery due to IBD**, (n, %)	
CD patients	
Bowel resection	5 (20.8)
Perianal disease	1 (4.2)
Liver transplantation due to PSC	1 (4.2)
UC patients	0 (0)
**Concomitant therapy for IBD**	
Corticosteroids, n (%)	1 (4.2)
AZA or MTX, n (%)	1 (4.2)
**Disease activity**	
HBI, n (median, minimum–maximum)	11 (0, 0–3)
PMS, n (median, minimum–maximum)	13 (0, 0–0)
Clinical remission, n (%)	24 (100)
CRP, mg/L, n (median, minimum–maximum)	23 (3.6, 0.4–23.1)
FC, µg/g, n (median, minimum–maximum)	16 (67, 16–772)
Biochemical remission, n (%)	16 (66.7)
**Serum vedolizumab trough concentration during IV therapy** (mg/L), mean (SD)	22.57 (15.42)

Abbreviations: IV—intravenous, CD—Crohn’s disease, UC—ulcerative colitis, IBD—inflammatory bowel disease, ASA—aminosalycilates, AZA—azathioprine, MTX—methotrexate, PSC—primary sclerosing cholangitis, HBI—Harvey–Bradshaw index, PMS—Partial Mayo score, CRP—C-reactive protein, FC—fecal calprotectin, SD—standard deviation.

**Table 2:** Change in vedolizumab trough concentration, fecal calprotectin level, C-reactive protein level, Harvey–Bradshaw index, and Partial Mayo score before and after transition from intravenous vedolizumab to subcutaneous vedolizumab.

Outcome	Baseline	After 6 Months	*p* Value
Vedolizumab serum trough concentration (mg/L), n = 22, mean (SD)	22.86 (15.66)	35.62 (15.46)	0.002 *
Fecal calprotectin (μg/g), n = 15, median (minimum–maximum)	67 (16–772)	58.5 (16–1230)	0.570 **
C-reactive protein (mg/L), n = 19, median (minimum–maximum)	3.6 (0.4–23.1)	6.8 (0.6–34.5)	0.126 **
Harley-Bradshaw index, n = 11, median (minimum–maximum)	0 (0–3)	0 (0–3)	0.317 **
Partial Mayo score, n = 13, median (minimum–maximum)	0 (0)	0 (0–4)	0.102 **

Abbreviations: SD—standard deviation. * Student’s *t*-test. ** Wilcoxon signed ranks test.

**Table 3:** Incidence of treatment-emergent adverse events by system organ class and preferred term.

Treatment-Emergent Adverse Events (n, %)	All Patients (N = 24)
Infections and infestations	5 (20.8)
COVID-19	4 (16.6)
Fungal foot infection	1 (4.2)
Neoplasms benign, malignant and unspecified (incl cysts and polyps)	2 (8.4)
Anogenital warts	1 (4.2)
Bowen’s disease	1 (4.2)
General disorders and administration site conditions	2 (8.4)
Pyrexia	1 (4.2)
Injection site erythema	1 (4.2)
Skin and subcutaneous tissue disorders	2 (8.4)
Urticaria	1 (4.2)
Pruritus	1 (4.2)
Blood and lymphatic system disorders	1 (4.2)
Iron deficiency anaemia	1 (4.2)
Musculoskeletal and connective tissue disorders	1 (4.2)
Arthritis	1 (4.2)

## Data Availability

Data is contained within the article.
